# Invasive wild boar (*Sus scrofa*) as a functional reservoir for the dynamics of *Trichinella* in the Patagonia region

**DOI:** 10.1590/S1984-29612024046

**Published:** 2024-09-09

**Authors:** Elizabeth Chang Reissig, Marcos Laugue, Graciana Gatti, Silvio Krivokapich

**Affiliations:** 1 Departamento de Recursos Naturales, Estación Experimental Agropecuaria Bariloche – EEA, Instituto de Investigaciones Forestales y Agropecuarias Bariloche – IFAB (INTA-CONICET), Instituto Nacional de Tecnología Agropecuaria – INTA, Bariloche, Argentina; 2 Consejo Nacional de Investigaciones Científicas y Técnicas – CONICET, Buenos Aires, Argentina; 3 Direccion General de Sanidad Animal y Fiscalización, Dirección Provincial de Sanidad y Emergencia Agraria, Subsecretaria de Producción, Ministerio de Producción e Industria, Gobierno de la Provincia del Neuquén, Neuquén, Argentina; 4 Departamento de Parasitología, Administración Nacional de Laboratórios e Institutos de Salud Dr. Carlos G. Malbrán, Instituto Nacional de Enfermedades Infecciosas - INEI, Buenos Aires, Argentina

**Keywords:** Wild boar, trichinellosis, public health, hunting, natural areas, invasive ungulate, Javali, triquinelose, saúde pública, caça, áreas naturais, ungulados invasores

## Abstract

Trichinellosis is a zoonotic disease that has been studied mainly in domestic pigs (*Sus scrofa domesticus*). The cycle involves infection in domestic and wild fauna, which fulfill complex ecological roles, where *Trichinella spiralis* is reported in wild boar (*Sus scrofa*). The objective of this study was to determine the prevalence of trichinellosis in wild boar and evaluate the distance of positive animals to the nearest urbanization areas in Argentina Patagonia. Necropsies were carried out on wild boar hunted in the Nahuel Huapi and Lanín National Parks and surrounding areas. Skeletal muscle samples were collected from 1,694 wild boar and artificial digestion was performed on all samples. *Trichinella* spp. were found in 96 (5.8%) wild boar (0.2 to 424 Larvae/g). Parasitism in wild boar depends on the distribution of the population in natural and urban areas. Infected wild boar were found near peri-urban areas, demonstrating the importance of routine epidemiological surveillance and sanitary measures in and around cities. More research is needed to identify the *Trichinella* species that infect wild animals. We recommend the application of active and passive epidemiological surveillance in South America on exotic and native fauna that are hunted and consumed by humans.

## Resumo

A triquinelose é uma doença zoonótica que tem sido estudada principalmente em porcos domésticos (*Sus scrofa domesticus*). O ciclo envolve infecção em fauna doméstica e silvestre, que cumprem papéis ecológicos complexos, onde *Trichinella spiralis* é reportada em javali (*Sus scrofa*). O objetivo deste estudo foi determinar a prevalência da triquinelose em javalis e avaliar a distância entre animais positivos em relação às áreas urbanas na Patagônia Argentina. Necropsias foram realizadas em javalis caçados nos Parques Nacionais Nahuel Huapi e Lanín e nas áreas circunvizinhas. Amostras de músculo esquelético foram coletadas de 1.694 javalis e a digestão artificial foi realizada em todas as amostras. *Trichinella* spp. foram encontrados em 96 (5,8%) javalis (0,2 a 424 Larvae/g). O parasitismo em javalis depende da distribuição da população em áreas naturais e urbanas. Javalis infectados foram encontrados perto de áreas periurbanas, demonstrando a importância da vigilância epidemiológica de rotina e de medidas sanitárias nas cidades e arredores. Mais pesquisas também são necessárias para identificar as espécies de *Trichinella* que infectam animais silvestres. Recomenda-se a aplicação de vigilância epidemiológica ativa e passiva, na América do Sul, sobre a fauna exótica e nativa que é caçada e consumida pelo ser humano.

## Introduction

Wild boar (*Sus scrofa*) is a species widely distributed around the world and is considered an important risk for the transmission of diseases to wildlife and humans (Novillo & Ojeda, 2008). In natural habitat, the species is considered omnivorous and generalist, and its diet included a variety of food, such as different types of flora, animal carrion, eggs and birds of ground-nesting, small mammals, and invertebrates (Garza et al., 2018). Invasive wild boar is considered a threat to the environment conservation, since they can disturb ecosystems, predate native fauna, and harbour zoonotic diseases as the trichinellosis (Pozio, 2007; Novillo & Ojeda, 2008).

*Trichinella* spp. is a nematode of worldwide distribution primarily of carnivorous and omnivorous mammals (Pozio, 2007). Wild mammals can act as natural reservoirs and have an important role in the propagation of the parasite. Currently, the *Trichinella* genus includes 10 species (*T. spiralis*, *T. nativa*, *T. britovi*, *T. pseudospiralis*, *T. murrelli*, *T. nelsoni*, *T. papuae*, *T. zimbabwensis*, *T. patagoniensis*, *T. chanchalensis*) and 3 genotypes (*Trichinella* T6, T8, and T9) (Zarlenga et al., 2020).

Domestic pigs contribute to the flow of *T. spiralis* by transmitting it to wild animals, while the different sylvatic species and genotypes of *Trichinella* cycle could be transmitted to from the wild to the domestic animals (Pozio, 2000; Ribicich et al., 2010, 2020). Synantropic fauna, such as rats (*Rattus norvegicus*), cats (*Felis silvestris catus*), and dogs (*Canis lupus familiaris*) are reservoirs of the parasite and important species in both cycles (Ribicich et al., 2010, 2020).

Trichinellosis is an endemic and emerging disease in Argentina (Pozio, 2001) and it has been reported in domestic pigs, wild animals, and in humans (Ribicich et al., 2020). The wild boar (exotic), pumas (*Puma concolor*), and armadillos (*Chaetophractus villosus*) have an important role in the maintenance of the sylvatic cycle in this country (Cohen et al., 2010; Krivokapich et al., 2012; Ribicich et al., 2020). The parasite can infect human through the consumption of raw or uncooked meat, mainly from domestic pig production. In Argentina, human cases of trichinellosis reached 6,662 suspicious cases during 2012-2018 (Ribicich et al., 2020). The disease has been recorded in different provinces, where the highest prevalence reported in the provinces San Luis, Córdoba, Mendoza, Santa Fé, and Buenos Aires (Ribicich et al., 2020). The zoonosis was also reported in Patagonian provinces of Argentina, such as Río Negro and Neuquén (Bolpe & Boffi, 2001; Di Pietro et al., 2004; Ribicich et al., 2020). In Chile, the first case of human trichinellosis was reported due to consumption of wild boar meat (García et al., 2005). To the best of our knowledge, the prevalence of trichinellosis in the Andean region has only scantily been investigated.

The aims of this study were to determine the infection and the prevalence of trichinellosis in wild boar that were hunted in the Nahuel Huapi and Lanin National Parks and surrounding areas at the Patagonia Argentina; and to compare positive *Trichinella* spp. wild boar in relation to distances from the hunting point and the location of the nearest and main Northwest Patagonian cities in Argentina.

## Material and Methods

### Study area

**Figure 1:**
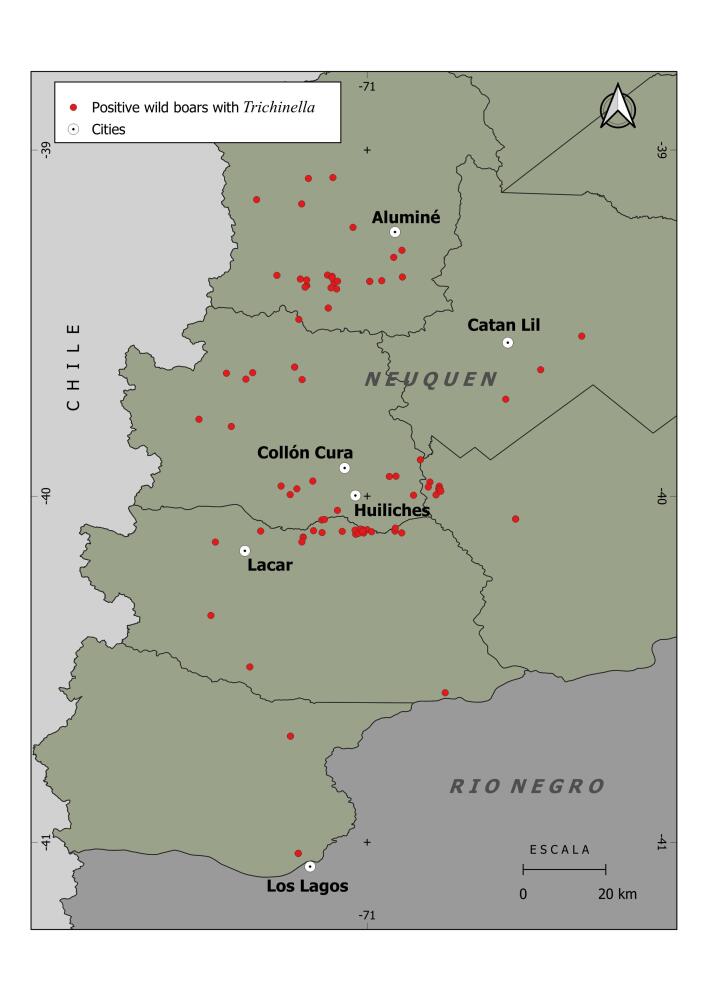
Study sites where wild boar positive for *Trichinella* were found along Northern Patagonia, Rio Negro and Neuquén Province, Argentina (2013 to 2017 hunting season). The main surrounding Patagonian cities are shown.

This study was carried out in hunting sites of the Nahuel Huapi National Park (NHNP), Lanin National Park (LNP), and in surrounding livestock farms located in the northwest Patagonian region of Argentina (Figure 1). The areas of national parks comprise 717,261 ha (NHNP) and 412,013 ha (LNP) of temperate forest, ecotone, and steppe. The vegetation is characterized by the predominance of native cypress forests of the Cordillera (*Austrocedrus chilensis*) and coihues (*Nothofagus dombeyi*), and steppes of mixed grasses (e.g. *Festuca*, *Poa* and *Stipa*) and shrubs (e.g. *Baccharis*,*Mulinum*, *Senecio*) with a coverage of 40 to 80% of the landscape. It also has forests of invasive trees such as conifers (*Pinus ponderosa*, *P. contorta* var. latifolia, *Pseudotsuga mensiezii*) and willows (*Salix* spp.) and shrubs dominated by *Rosa rubiginosa*). The main activity in the private farms is extensive cattle and sheep farming, with wool and meat production purposes. There is a strong tourist and hunting activity of exotic ungulates, such as wild boar and red deer (*Cervus elaphus*). San Carlos de Bariloche, Villa La Angostura, San Martín de los Andes, and Junín de los Andes are the main cities located nearby and surrounding the areas of the national parks.

### Sampling collection

Skeletal muscle samples (tongue, intercostal, diaphragm, hindquarters) of wild boar were collected during 2013 to 2017 hunting season of the NHNP and LNP. Samples were collected by local veterinarians who received the samples in the field directly from the hunters, and by one of the authors of this paper (ECR) in a processing plant for smoked wild boar in San Carlos de Bariloche city, Los Lagos region, Northern Patagonia. Skeletal muscle samples were collected and frozen at – 20ºC.

### Artificial digestion and PCR analysis

Muscle samples were examined by the standard artificial digestion method (Gamble et al., 2000). DNA was extracted from single muscle larvae of *Trichinella* in accordance with a published protocol (Krivokapich et al., 2006). Identification to the species level was made by a multiplex PCR of nuclear ribosomal DNA (Zarlenga et al., 1999).

### Statistical analysis

Wild boar were classified according to geographic location (region and locality of hunting sites), with only positive specimens included. Statistical analyses were performed to compare the distances between hunting sites of positive animals and closer Patagonian cities (San Carlos de Bariloche, Villa La Angostura, San Martín de los Andes, and Junín de los Andes).

## Results

**Figure 2:**
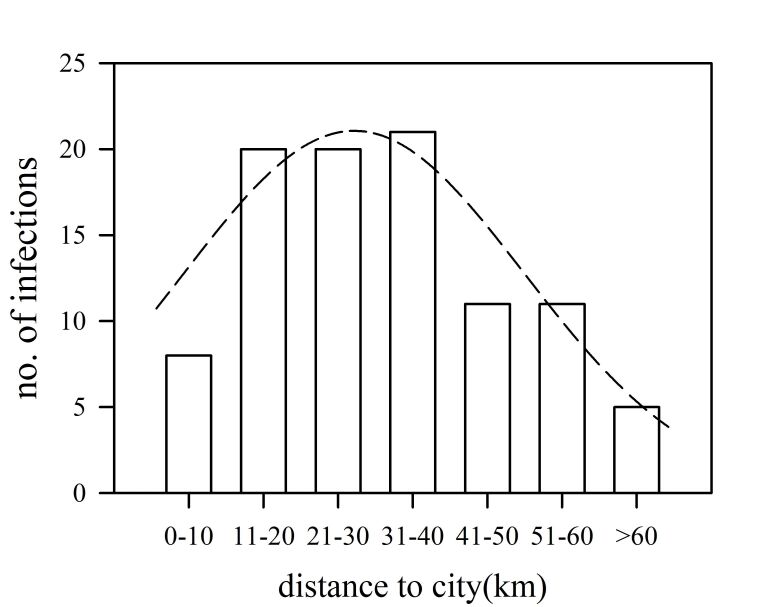
Relationship between no. of infected wild boar hunted in Northwest Patagonia and the distance to the nearest urbanization. Dash line represents the fitted Gaussian model between variables as follows: $y=a\;e^{{\left[(-0.5\;\left(\frac{\left(x-x0\right)}{b}\right)\right]}^{2}}$ , where*y*and*x*are the main variables and model parameters are: a = 21.3, b = 19.2, x_0_= 32.9, r^2^= 0.94, P < 0.0001.

Larvae of *Trichinella* were found in 96 out of 1694 (5.8%) wild boar (0.2 to 424 Larvae/gr) using the artificial digestion method. From those positive samples, one isolates were identified as *T. spiralis* by multiplex PCR technique. In most of the samples was not possible to perform molecular identification due to the difficulty to deliver muscle samples from field sites to the laboratory at the Buenos Aires city. The number of wild boar infected with *Trichinella* spp. tended to be higher near towns and cities in the study area, with maximum numbers of infected animals found (more than 20) at a distance of 11 to 40 km to urban spots (Figure 2). At the landscape level, the highest number of infected wild boar were hunted along the transition (ecotone) between Andean Forest and steppe, where cities are settled.

## Discussion

During the last few years, there has been an increase in the wild populations of wild boar in Europe and America and a growing concern about the diseases they can transmit, mainly to humans (Ruiz-Fons et al., 2008). Several etiological agents reported in wild boar and the role of this species as a natural reservoir is currently being discussed (Ruiz-Fons et al., 2008). Serological surveys have been carried out on feral pig, wild and captive wild boar populations to assess their health status and detect the prevalence of infectious-contagious diseases of zoonotic and economic concern. However, to the best of our knowledge, many of them have not been sufficiently studied, mainly in the southern cone of South America where an increase of wild boar population in natural areas has been reported (Novillo & Ojeda, 2008; Ballari et al., 2016).

In Patagonia region, *Trichinella* spp. outbreaks has been suspected in humans following consumption of infected raw meat products made from domestic pig farming or culled wild boar (Ribicich et al., 2020). In this study, trichinellosis infection in free-range wild boar shows a low prevalence in animals hunted in ecotone environment of Patagonia Argentina. Positive wild boar by artificial digestion technique were identify as *T. spiralis* despite other sylvatic *Trichinella* species, such as *T. patagoniensis*, were previously reported in wild patagonia fauna (Krivokapich et al., 2008, 2012). In the El Palmar National Park, northern Argentina, similar results showed the circulation of *T. spiralis* in wild boar with a low parasite burden and suggest the influence of biosecurity practice to reduce the transmission (Cohen et al., 2010; Tammone Santos et al., 2024).

At present, trichinellosis is considered an emerging disease due to the increase of its prevalence in countries such as Argentina, Bulgaria, China, Croatia, Lithuania, Mexico, Romania, Russia, and former Yugoslavia (Pozio et al., 1996; Pozio, 2001, 2007; Ribicich et al., 2010). The trichinellosis human cases in Argentina are associated with the increase in pork consumption and may have also regarding with the wild boar hunting activity along the country, (Ribicich et al., 2020). Although the wild boar can harbour various zoonotic pathogens, including parasitic diseases of public health importance (Fernandez-de-Mera et al., 2003; Solaymani-Mohammadi et al., 2003; Solaymani-Mohammadi & Petri, 2006), few studies have been conducted on this European exotic and invasive ungulate in South America. Apparently, the prevalence of trichinellosis is moderate in Argentina, however, human cases continue to occur due to consumption of meat and derivatives of infected pigs and boars, without a proper food security inspection during slaughter (Di Pietro et al., 2004; Ribicich et al., 2020).

Trichinellosis poses a widespread concern across Argentina, with infections documented in both domestic and wild animals, as well as in humans (6,662 human cases from 2012 to 2018, Ribicich et al., 2020). The parasite, *Trichinella*, has been identified in various species including pigs, wild boars, dogs, cats, rats, armadillos, pumas, opossums (*Didelphis albiventris*), and even South American sea lions (*Otaria flavescens*) (Krivokapich et al., 2006; Cohen et al., 2010; Krivokapich et al., 2012; Ribicich et al., 2020). In 2022, patagonia Argentina provinces (our study area) have a human population of 750,768 in Río Negro (3,5 hab./km^2^), and 726,590 in Neuquén (7,2 hab./km^2^). These provinces in 2012-2018 reported 39 to 231 trichinellosis human cases, where domestic pig, wild boar and puma were the main hosts involved in the infections (Ribicich et al., 2020). This broad spectrum of hosts underscores the extensive reach of the parasite within the country, highlighting the importance of vigilant monitoring and preventive measures to safeguard public health.

The *Trichinella* spp. have a domestic and sylvatic cycle, the latter related to the distribution of the reservoirs in the natural habitat and their interaction with predators and scavengers (Pozio, 2000, 2007; Ribicich et al., 2020). Association between rats and pigs was reported as a risk factor for trichinellosis infection, and it is known that sanitary measures in pig production reduce the occurrence of the disease (Ribicich et al., 2010). However, recently, a new species of *Trichinella* (*T. patagoniensis*) has been identified in puma (Krivokapich et al., 2012) and different species of *Trichinella* were found in Argentina (Krivokapich et al., 2015, 2019; Ribicich et al., 2020). Little is known about the sylvatic cycle of *Trichinella* in wildlife and the role that exotic ungulates play in the transmission of the parasite. This study shows that wild boar introduced in Argentina can act as reservoirs in the maintenance and dissemination of trichinellosis as native fauna do (predators and other scavengers), and even in low prevalence as finding here, can stand as a public health issue by infecting the local rural community (farmers, hunters, guide-hunters) and urban people (city residents and tourists).

Wild boar population control and eradication are difficult to carry out in Patagonia Argentina. The species is invasive, the population is growing and widely distributed with no limit in its movements (Ballari et al., 2016). In previous studies, animals regularly move between hunted sites, private or protected areas, and shown a home-range from 0.62 to 34.4 km for females and 1.05 to 48.34 km for males, with no significant differences between both sex (Garza et al., 2018). In Argentina, exotic wild boar lacks effective natural predators in natural landscape, and even having the hunting season allowed through the year in the Patagonia provinces, no population reduction is reached. It has been studied that home-range increased in areas with greater native mammal’s richness (Garza et al., 2018), however, wild boar population density and animal movement has been little analyzed in Patagonia.

Trichinellosis is considered an endemic parasitosis in Argentina. Among the provinces there are endemic areas of human and animals infected (Ribicich et al., 2020). This work shows that the parasitic disease is an important public health problem in the country. In our study area, positive wild boar were found close to urban areas (Figure 2); the distance reported of infected wild boar hunted were from 11 to 40 km close to cities. Therefore, implementation of wild boar population control near urban sites, and direct diagnostic of *Trichinella* are needed to prevent trichinellosis propagation and infection in Patagonia region. Moreover, it will be of concern to improve the efficacy of food chain, biosecurity, and veterinary inspection on hunting sites, as well as regulating the commercial manufacture of wild boar products, and reinforce zoonotic diseases education program to local Patagonian community.
